# From the ultrasonic to the infrared: molecular evolution and the sensory biology of bats

**DOI:** 10.3389/fphys.2013.00117

**Published:** 2013-05-30

**Authors:** Gareth Jones, Emma C. Teeling, Stephen J. Rossiter

**Affiliations:** ^1^School of Biological Sciences, University of BristolBristol, UK; ^2^UCD School of Biology and Environmental Science, University College DublinDublin, Ireland; ^3^School of Biological and Chemical Sciences, Queen Mary, University of LondonLondon, UK

**Keywords:** echolocation, hearing, vision, olfaction, taste, perception

## Abstract

Great advances have been made recently in understanding the genetic basis of the sensory biology of bats. Research has focused on the molecular evolution of candidate sensory genes, genes with known functions [e.g., olfactory receptor (OR) genes] and genes identified from mutations associated with sensory deficits (e.g., blindness and deafness). For example, the *FoxP2* gene, underpinning vocal behavior and sensorimotor coordination, has undergone diversification in bats, while several genes associated with audition show parallel amino acid substitutions in unrelated lineages of echolocating bats and, in some cases, in echolocating dolphins, representing a classic case of convergent molecular evolution. Vision genes encoding the photopigments rhodopsin and the long-wave sensitive opsin are functional in bats, while that encoding the short-wave sensitive opsin has lost functionality in rhinolophoid bats using high-duty cycle laryngeal echolocation, suggesting a sensory trade-off between investment in vision and echolocation. In terms of olfaction, bats appear to have a distinctive OR repertoire compared with other mammals, and a gene involved in signal transduction in the vomeronasal system has become non-functional in most bat species. Bitter taste receptors appear to have undergone a “birth-and death” evolution involving extensive gene duplication and loss, unlike genes coding for sweet and umami tastes that show conservation across most lineages but loss in vampire bats. Common vampire bats have also undergone adaptations for thermoperception, via alternative splicing resulting in the evolution of a novel heat-sensitive channel. The future for understanding the molecular basis of sensory biology is promising, with great potential for comparative genomic analyses, studies on gene regulation and expression, exploration of the role of alternative splicing in the generation of proteomic diversity, and linking genetic mechanisms to behavioral consequences.

## Introduction

Bats perceive the world by using a wide range of sensory mechanisms, some of which have become highly specialized (Altringham and Fenton, 2003). Vision is ineffective in complete darkness (although many pteropodids rely largely on vision in dimly lit conditions); hence most bats use echolocation for orientation, and often for prey detection and localization. The literature on the sensory biology of bats is therefore dominated by research on echolocation (Griffin, 1958; Thomas et al., 2004; Jones, 2005). Echolocation is now understood in depth from neurobiological mechanisms (Pollak and Casseday, 1989; Popper and Fay, 1995) through to behavioral and ecological correlates of signal design (e.g., Kalko and Schnitzler, 1998; Schnitzler and Kalko, 1998; Jones and Holderied, 2007). Bats use ultrasound and lower frequency sound for communication, and have evolved rich repertoires of social calls (e.g., Clement et al., 2006; Ma et al., 2006; Bohn et al., 2009; Carter et al., 2012). Considerable advances are being made to understand the role of sound in communication (Jones and Siemers, 2011; Puechmaille et al., 2011). In contrast, the roles of others senses in the lives of bats are less well-understood, even though these senses can be of fundamental importance. Ecological aspects of vision, olfaction, touch, and thermoperception are reviewed by Altringham and Fenton (2003) who concluded that “*with some notable exceptions, our knowledge about vision and olfaction has not advanced greatly since Suthers's (1970) review, compared to the enormous strides made in studies on echolocation*.” This stems partially from the great difficulty in observing and measuring these senses in wild, nocturnal flying mammals such as bats.

Recent years have seen considerable progress in our understanding of the genetic basis of sensory perception, attributable in part to advances in molecular genetics technologies and the associated abundance of new comparative sequence data. Most recent work has focussed on “candidate genes” associated with specific sensory traits. Candidate genes are genes known to be involved in pathways that affect phenotypes; sequencing these in individuals with unusual or different phenotypes can help identity mutations that can be related to adaptation (Stapley et al., 2010). For example, sequencing genes that possess mutations associated with non-syndromic deafness in humans has been valuable in identifying genes likely to be important in audition in other mammals, including bats, and understanding the molecular adaptations and mutations associated with auditory specialization and disease predisposition (Kirwan et al., 2013). One of the aims of this paper is to review studies on candidate genes associated with sensory perception in bats, and to show how these studies have elucidated our understanding of evolutionary processes, especially positive selection, convergent evolution and sensory trade-offs in which specialization in one sensory modality may result in reduced neural (and consequently genetic) investment in other senses (Harvey and Krebs, 1990). The identification of candidate genes is a first step in elucidating molecular mechanisms underpinning the sensory biology of bats.

In this paper we review advances in our knowledge of the genetic basis of sensory behavior in bats. We consider echolocation at the levels of both signal production and reception. We then describe how sequencing studies of genes associated with vision, olfaction, taste and thermoperception have revealed remarkable cases of convergent evolution, sensory trade-offs and novel adaptations. Gene symbol nomenclature is dynamic, and in this review we have followed the symbols used by the authors of the research papers on bats, though always presenting the symbols in lower case as is recommended for non-human homologues. Some of these gene symbols differ from those in the official nomenclature (see www.genenames.org), and the symbols used in the original papers on bats are listed alongside the official gene symbols and the approved gene names can be determined from Table 1. With molecular methods advancing rapidly, we conclude by outlining approaches that can potentially build on findings from candidate gene studies. We conclude by considering future opportunities for further developing this field, which has been one of the most fast-moving and exciting in research on bats in recent years.

**Table 1:** **Genes referred to in the text**.

**Sense**	**Gene symbol**	**Approved name**
Echolocation	*FoxP2*	Forkhead box P2
	*Slc26a5 (Prestin)*	Solute carrier family 26, member 5 (Prestin)
	*Kcnq4*	Potassium voltage-gated channel, KQT-like subfamily, member 4
	*Tmc1*	Transmembrane channel-like 1
	*Dfnb59 (Pjvk)*	Deafness, autosomal recessive *59*
	*Cdh23*	Cadherin-related 23
	*Pcdh15*	Protocadherin-related 15
	*Otof*	Otoferlin
	*Wnt8a*	Wingless-type MMTV integration site family, member 8A
	*Fos*	FBJ murine osteosarcoma viral oncogene homolog
	*Chrna10*	Cholinergic receptor, nicotinic, alpha 10 (neuronal)
	*Myo15A (Myo15)*	Myosin XVA
	*Ush1g*	Usher syndrome 1G (autosomal recessive)
	*Strc*	Stereocilin
	*Tectb*	Tectorin beta
	*Otog*	Otogelin
	*Col11a2*	Collagen, type XI, alpha 2
	*Gjb2*	Gap junction protein, beta 2, 26kDa
	*Cldn14*	Claudin 14
	*Pou3f4*	POU class 3 homeobox 4
	*Myo6*	Myosin VI
Vision	*Rh1*	Rhodopsin
	*Crx*	Cone-rod homeobox
	*Sag*	S-antigen; retina and pineal gland (arrestin)
	*Opn1sw (SWS1)*	Opsin 1 (cone pigments), short-wave-sensitive
	*Opn1mw (M/lws)*	Opsin 1 (cone pigments), medium-wave sensitive
Olfaction	*OR*	Used to refer to the family of olfactory receptor genes
	*Trpc2*	Transient receptor potential cation channel, subfamily C, member 2
Taste	*Tas1r1*	Taste receptor, type 1, member 1
	*Tas1r2*	Taste receptor, type 1, member 2
	*Tas1r3*	Taste receptor, type 1, member 3
Thermoperception	*Trpa1*	Transient receptor potential cation channel, subfamily A, member 1
	*Trpv1*	Transient receptor potential cation channel, subfamily V, member 1

Nomenclature follows HUGO Gene Nomenclature Committee (www.genenames.org). Names used in papers cited in the text are given in brackets after the approved gene name. Approved names are for human genes, except for Trpc2 where the gene has become pseudogenized in humans where the mouse homologue (Mouse Genome Informatics—www.informatics.jax.org) is listed.

## Echolocation

**Figure 1:**
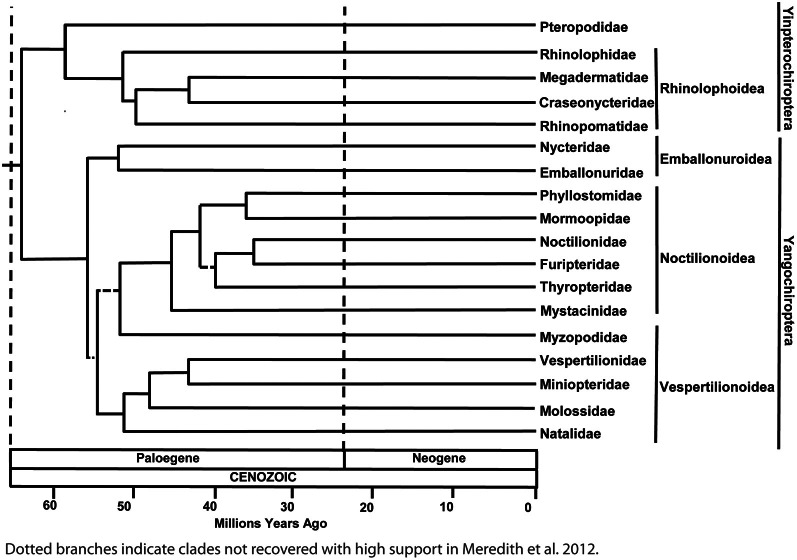
**Most recent phylogenetic arrangement of bat families based on dating and consensus analysis of amino acid and DNA analyses.** Dotted lines represent branches not recovered with high support. See Meredith et al. (2011) for details. A newly suggested family, Cistugidae, would be basal to the Vespertilionidae in this tree, having diverged from this family approximately 35 MYA (Lack et al., 2010). Reproduced with permission from the American Association for the Advancement of Science.

To better understand the implications of molecular studies for the evolution of echolocation, it is necessary to appreciate the current view on phylogenetic relationships among bat families. Evidence from a wide range of gene sequencing studies supports the hypothesis that bats using laryngeal echolocation (i.e., which produce signals in the larynx) are paraphyletic. Bats in the family Pteropodidae do not use laryngeal echolocation (though bats in one genus—*Rousettus*—echolocate by tongue clicking), but belong to the suborder Yinpterochiroptera that also includes laryngeal echolocators from the families Megadermatidae, Craseonycteridae, Rhinopomatidae, Hipposideridae, and Rhinolophidae (Teeling et al., 2005; Meredith et al., 2011). Some of these bats, notably the horseshoe bats (Rhinolophidae) and Old World leaf-nosed bats (Hipposideridae) arguably possess the most sophisticated echolocation systems known of all organisms. Indeed the close evolutionary relationship between the Pteropodidae and the families Rhinolophidae and Hipposideridae is surprising given that the latter have a particularly specialized sonar involving the emission of long constant frequency (CF) calls permitting the classification of insect prey, combined with broadband sweeps for localizing targets and the ability to adjust the frequency of emitted calls to compensate for Doppler shifts induced by their flight speed (Schnitzler, 1968; Trappe and Schnitzler, 1982; Hiryu et al., 2005). All the other 15 families of bats that use laryngeal echolocation, including the recently proposed Miniopteridae (see Miller-Butterworth et al., 2007) and Cistugidae (see Lack et al., 2010), are classified in the suborder Yangochiroptera (Figure 1; see also Teeling et al., 2000, 2005; Jones and Teeling, 2006; Meredith et al., 2011).

**Figure 2:**
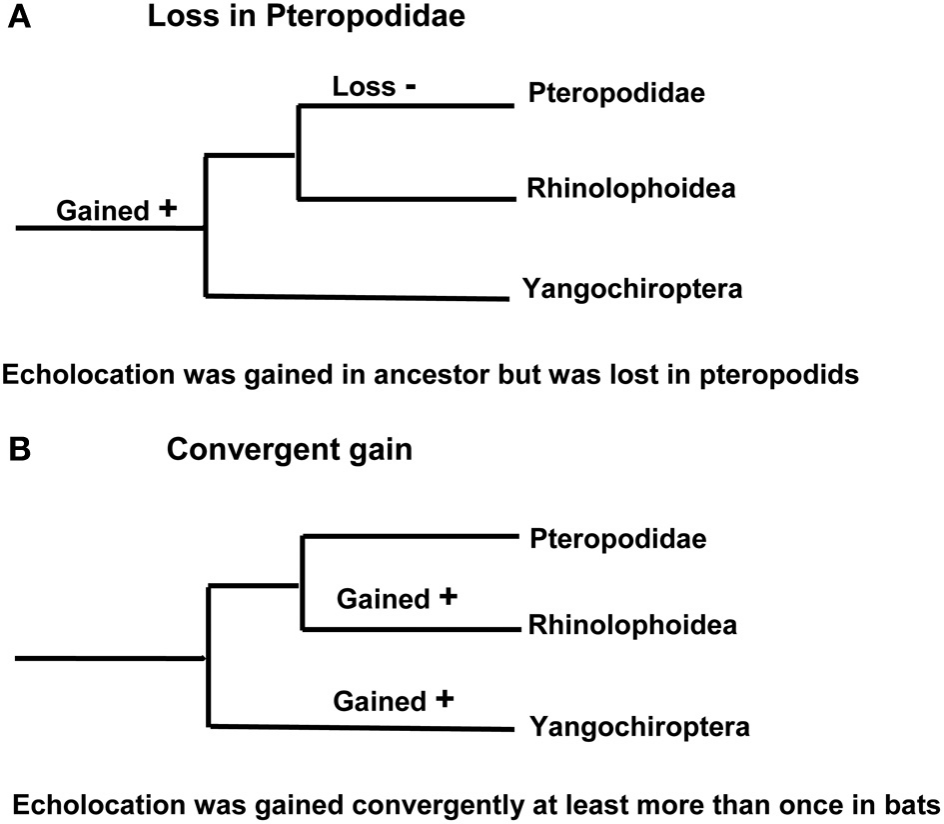
**Alternative hypotheses for the evolution of laryngeal echolocation. (A)** Phylogenetic tree showing a single loss in the Old World fruit bats. **(B)** Phylogenetic tree in which echolocation was acquired independently by more than one lineage.

This phylogenetic arrangement of bats raises two alternative scenarios about the evolution of laryngeal echolocation. Either echolocation had evolved in the common ancestor of all extant bats, and was subsequently lost in the Pteropodidae [with echolocation evolving secondarily by tongue-clicking in cave roosting bats in the genus *Rousettus* (Möhres and Kulzer, 1956; Yovel et al., 2011)], or echolocation evolved independently (possibly even on several occasions) in the Yinpterochiroptera and the Yangochiroptera (Figure 2). It seems reasonable to assume that molecular genetic analyses should be helpful in discriminating between these hypotheses: the independent evolution of echolocation may have resulted in different genetic mechanisms being recruited for echolocation in different lineages of bats, while a single origin predicts that extremely similar genetic mechanisms will underpin echolocation in all bats and molecular loss-of-function should be evident in the pteropodids (predictions reviewed in Teeling et al., 2012). Anatomical evidence suggests that several bat species known from fossils in the Eocene were likely to have used echolocation, hence the ability to echolocate has been present in most bats during all of their known fossil history (Simmons et al., 2008; Teeling, 2009a; Teeling et al., 2012).

Reviews of some of the candidate genes likely to be involved in echolocation have been conducted by Maltby et al. (2009), Teeling (2009b), and Teeling et al. (2012), and the reader is referred to these for more detail.

### Vocalization

*FoxP2* is a gene coding for a transcription factor associated with vocalizations and sensory-motor integration. Briefly, mutations in *FoxP2* affect production and comprehension of language in humans (see review by Fisher and Marcus, 2006) and two adaptive substitutions in *FoxP2* that occurred since humans split from a common ancestor with chimpanzees suggest that *FoxP2* was important in the evolution of human language (Enard et al., 2002). Although *FoxP2* is highly conserved in most mammals studied, it shows high levels of diversity, as well as evidence of divergent selection, in echolocating bats (Li et al., 2007; Zhang et al., 2013). Li et al. (2007) found exons 7 (likely to be important in the evolution of language in humans) and 17 to be especially divergent in bats compared with other mammals, and a recent whole-genome analysis detected even higher divergence in Exon 3 of *FoxP2* in *Myotis davidii* compared with the mammalian consensus sequence (Zhang et al., 2013). Because echolocation involves vocal behavior and extreme sensory-motor coordination it seems likely that the accelerated evolution of *FoxP2* in echolocating bats is related to the evolution of diverse types of echolocation strategies and their integration with subsequent motor behavior such as manoeuvring in flight (Li et al., 2007). However, molecular evolutionary analyses of two highly variable exons in *FoxP2* did not provide unequivocal insights into whether laryngeal echolocation evolved on more than one occasion in bats (also see Teeling et al., 2012). Moreover, to date there is no clear reason for the variation seen in *FoxP2* in bats. Examination of existing genome data suggests this gene is present as a single copy and, therefore, we can rule out duplication and neofunctionalization as a potential source of diversification. One explanation might be that *FoxP2* was recruited into the pathways underpinning echolocation early in the evolution of bats, and that observed sequence variation simply reflects the fact that echolocation is itself a highly variable trait that has undergone considerable divergence and convergence over the course of tens millions of years.

Gene silencing of *FoxP2* by lentivirus-mediated RNA interference is feasible (Chen et al., 2013), and opens opportunities for direct tests of whether *FoxP2* expression affects echolocation behavior in bats. Knockdown experiments show how *FoxP2* in the basal ganglia nucleus area X is important for accurate vocal imitation in birds (Haesler et al., 2007). Working with the CF echolocating bat, *Hipposideros armiger*, Chen et al. (2013) substantially reduced the typically high levels of *FoxP2* expression in the anterior cingulate cortex (ACC) of the brain, an area involved in motor control and important in vocalization (Paus, 2001). *FoxP2* silencing disrupts Doppler shift compensation in *H. armiger* confirming that it plays an important role in echolocation (Metzner and Schuller, 2009; Metzner and Zhang, 2009). These studies also found that *FoxP2* expression was higher in the suprageniculate nucleus and the ACC in the brains of bat species that use laryngeal echolocation (*Rhinolophus ferrumequinum*, *H. armiger* and *Myotis ricketti*), whereas in species without laryngeal echolocation (*Rousettus leschenaultii* and *Cynopterus sphinx*) expression was stronger in the olfactory tubercles (Metzner and Schuller, 2009; Metzner and Zhang, 2009). The identification of downstream neural targets affected by FoxP2 in bats remains as an exciting challenge; attempts to identify these binding targets in human neuron-like cells have revealed that FOXP2 either represses or activates gene expression at promoter sites involved in the modulation of synaptic plasticity, neurodevelopment, neurotransmission, and axon guidance (Vernes et al., 2007, 2011).

### Hearing

A number of recent studies have focussed on candidate genes associated with audition. The membrane motor protein Prestin drives mechanical amplification of sound in the outer hair cells (OHCs) of the cochlea. Prestin functions by directly converting voltage to displacement and consequently acts several orders of magnitude faster than enzymatically-driven proteins (Zheng et al., 2000). Knockout studies of mice suggest that Prestin may enhance auditory sensitivity 100-fold (i.e., by 40 dB) by electromotility resulting from its mechanical elongation and contraction (Liberman et al., 2002). Molecular evolutionary studies identified positive selection acting on anion-transporter genes in the *Slc26* family, resulting in the evolution of the *Prestin* gene (formally known as *Slc26a5*) on the evolutionary branch leading to mammals: subsequently *Prestin* has been under strong purifying selection in many mammalian lineages (Franchini and Elgoyhen, 2006).

**Figure 3:**
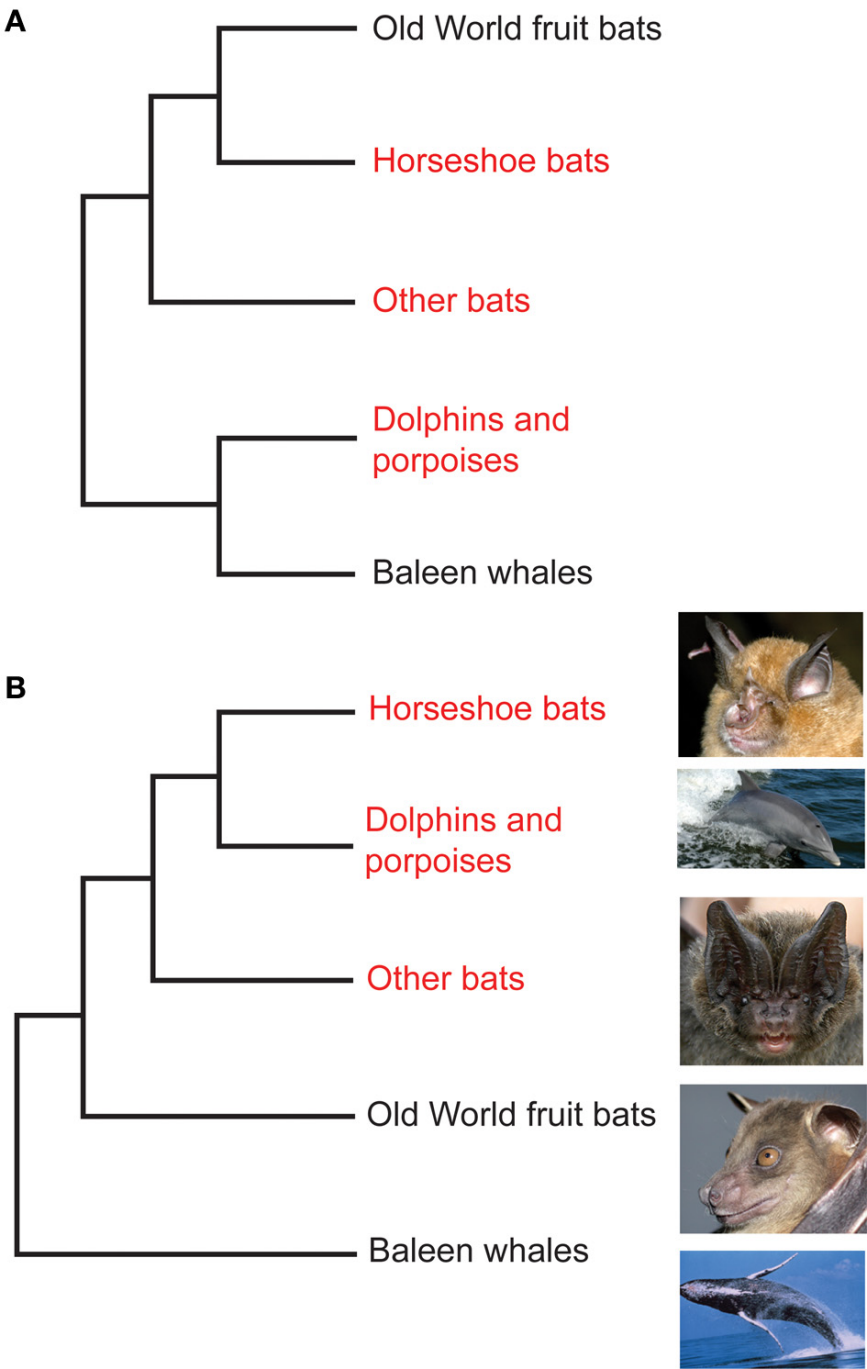
**Convergent evolution of prestin sequences in echolocating bats and cetaceans. (A)** Phylogeny of Old World fruit bats (Pteropodidae), horseshoe bats and their close relatives, other bat lineages studied by Liu et al. (2010a,b) and Li et al. (2010), dolphins and porpoises, and baleen whales as determined from large-scale molecular sequence analyses. **(B)** The arrangement that arises from analysis of the *Prestin* gene. Bat groups highlighted in red use laryngeal echolocation and cetacean groups highlighted in red exhibit biosonar behavior. In **(A)** these echolocating taxa are paraphyletic—non-echolocating Old World fruit bats are sister to echolocating horseshoe bats, and echolocating dolphins and porpoises are sister to non-echolocating baleen whales. In **(B)** all echolocating taxa form a monophyletic group, and dolphins and porpoises are the sister group of horseshoe bats. Photographs are species studied by Liu et al. (2010a,b) and Li et al. (2010). From top to bottom they are the greater horseshoe bat *Rhinolophus ferrumequinum* (G. Jones), the bottlenose dolphin *Tursiops truncatus* (NASA), the Beijing barbastelle *Barbastella beijingensis* (J. R. Flanders), the greater short-nosed fruit bat *Cynopterus sphinx* (G. Jones), and the humpback whale *Megaptera novaeangliae* (NOAA). From Jones (2010) reproduced with permission from Elsevier.

Phylogenetic tree reconstructions based on *Prestin* amino acid sequences recover an erroneous monophyletic group containing echolocating Yinpterochiroptera and Yangochiroptera lineages, rather than the accepted species tree in Figure 1 (Li et al., 2008). This startling result, coupled with the absence of any detectable relaxed selection acting on *Prestin* in non-echolocating fruit bats, suggests that the Prestin protein may have evolved convergently in echolocating lineages. More recently, *Prestin* sequences from echolocating bats and dolphins have also been found to contain convergent amino acid residues (Li et al., 2010; Liu et al., 2010a,b), (Figure 3) and appear to be concentrated in areas of the protein involved in voltage sensing (Li et al., 2010). In total, Liu et al. (2010a) found 10 amino acid sites in *Prestin* that appear to have evolved convergently in echolocating rhinolophoid bats and toothed whales providing one of the most compelling examples of convergent sequence evolution yet described (see Christin et al., 2010 for a review of other cases).

Positive selection acting on *Prestin* was also detected in rhinolophoid bats that use Doppler shift compensation and which emit calls with long CF components (Li et al., 2008). Prestin confers auditory selectivity as well as enhancing sensitivity (Zheng et al., 2002), and this is probably important for bats that use Doppler shift compensation as they possess especially sharp hearing (auditory foveae) to separate pulses from echoes by frequency and enhance the detection of fluttering targets (Schnitzler and Denzinger, 2011). Although the moustached bat, *Pteronotus parnelli* (Mormoopidae), from the New World has independently evolved an echolocation system that uses long CF signals and Doppler shift compensation (DSC), it shares most amino acid changes in Prestin with its congeners and with phyllostomid bats that do not use DSC rather than with rhinolophoid bats (Shen et al., 2011). Hence the adaptive changes found in *Prestin* of rhinolophid bats are not necessary for CF echolocation and DSC in *P. parnelli*, and different evolutionary trajectories in *Prestin* evolution occur for this specialized form of echolocation.

Positive selection acting on *Prestin* in rhinolophid bats that use DSC could result from the extreme selectivity used in auditory processing by these bats, or could arise because these bats emit calls with relatively high frequencies relative to their body size (Jones, 1999). The extent of protein evolution appears to be linked to the evolution of high-frequency hearing (Rossiter et al., 2011). In particular, there are more non-synonymous mutations in *Prestin* in whale and bat species that emit higher frequency vocalizations (and are therefore assumed to be more sensitive to higher frequencies), and in toothed whales, and the relationship remains even after accounting for phylogenetic relatedness (Liu et al., 2010b).

The gene *Kcnq4* encodes a protein that acts as a voltage-gated potassium channel involved in the regulation of electrical signaling. It is expressed in the OHCs, especially at the basilar part of the cochlea (Kharkovets et al., 2000). Mutations in *KCNQ4* in humans can cause the progressive loss of high frequency hearing (Kharkovets et al., 2006) hence its evolution in bats is of especial interest. The molecular evolution of *Kcnq4* in bats shows several parallels with patterns seen in *Prestin*. Echolocating bats form a monophyletic group in the *Kcnq4* nucleotide and amino acid sequence trees, and five amino acid sites are shared between echolocating bats in both suborders [Yinpterochiroptera and Yangochiroptera (Liu et al., 2011)]. Reconstruction of ancestral sequences suggests that bats in the two suborders evolved mutations at two amino acid sites in parallel. Moreover the number of amino acid replacements is positively correlated with assumed frequency of best hearing in both the Yangochiroptera and the Rhinolophoidea (Liu et al., 2011).

Liu et al. (2012) independently confirmed the monophyly of bats that use laryngeal echolocation in gene trees based on Kcnq4 amino acid (but not nucleotide) sequences, and identified eight shared substitutions among lineages that may have evolved under parallel evolution. Surprisingly, none of the eight parallel substitutions identified by Liu et al. (2012) match those identified by Liu et al. (2011). Again, the arguments for parallel evolution were developed in part because there was no evidence for relaxed selection acting on *Kcnq4* during the evolution of Old World fruit bats that do not use laryngeal echolocation.

Mutations in the genes *Tmc1* and *Pjvk* (formally known now as *Dfnb59*) result in non-syndromic hearing loss in mammals. *Tmc1* encodes a transmembrane protein found in inner and OHCs in the cochlea, and may function in moving molecules to the plasma membrane, or may provide intracellular regulatory signals during hair cell development (Marcotti et al., 2006). *Pjvk* encodes the protein pejvakin, and mutations in the gene cause auditory neuropathy in humans and vestibular defects in mice (see Davies et al., 2011). As is the case with *Prestin*, phylogenetic trees based on coding sequences of both genes group echolocating bats as a monophyletic clade (Davies et al., 2011). Some genetic convergence between whales and bats that use echolocation is also apparent (Davies et al., 2011). Convergent amino acid changes in bat clades that use high-frequency signals in echolocation support the hypothesis that both genes may be associated with high-frequency hearing, and parallel mutations in *Tmc1* shared between *R. ferrumequinum* and *P. parnellii* imply convergent evolution associated with CF echolocation and DSC in this case (Davies et al., 2011).

Although much research has focussed on genes involved in voltage motility, Shen et al. (2012a) investigated genes (*Cdh23* and *Pcdh15*) associated with hair bundle motility in OHCs, and *Otof*, which encodes a protein that may trigger membrane fusion in ribbon synapses in inner hair cells and potentially functions in transmitting auditory signals to the brain. Mutations in all these genes are again associated with deafness in humans. *Otof* shows strong expression in the auditory cortex of adult bats that use laryngeal echolocation (*Miniopterus schreibersii*) compared with *Rousettus leschenaultii* that echolocates by tongue clicking [which is a sophisticated but non-laryngeal form of echolocation (Yovel et al., 2011)]. Parallel evolution in all three genes was suggested for three groups of echolocating mammals (Yinpterochiroptera, Yangochiroptera, and toothed whales) (Shen et al., 2012a). The authors suggest that parallel evolution has hence occurred in a number of auditory processes—voltage motility, cochlear amplification and neural transduction—and that the processing of echolocation signals involved coevolution of genes that are involved in a number of pathways during auditory processing. It is remarkable that multiple genes involved in different auditory processes have shown independent evolution in three groups of echolocating mammals (Shen et al., 2012a). Recent sequencing of the genomes of an echolocating and a non-echolocating bat (Zhang et al., 2013) suggested that further echolocation-related genes include *Wnt8a* and *Fos*.

Despite these above findings, it is important to emphasize that cases of sequence convergence in which substitutions lead to erroneous phylogenetic groupings are still rare and most genes, including hearing genes, are expected to recover the recognized species tree. Liu et al. (2012) analysed the molecular evolution of *Chrna10*, a gene that encodes the α10 nicotinic acetylcholine receptor subunit important role for mediating synaptic transmission between medial olivocochlear fibers and OHCs, and for the inhibition of somatic electromotility (Elgoyhen et al., 2001). Trees based on *Chrna10* amino acid sequences resembled the species trees rendering bats that use laryngeal echolocation paraphyletic (Liu et al., 2012). Kirwan et al. (2013) undertook phylogenetic and selection analyses of 11 genes implicated in hearing (*Myo15 (Myo15a)*, *Ush1g*, *Strc*, *Tecta*, *Tectb*, *Otog*, *Col11a2*, *Gjb2*, *Cldn14*, *Kcnq4* [which was reported as showing parallel evolution by Liu et al. (2012)], *Pou3f4*) and found good support for the paraphyly of echolocating bats across these loci as well as a high level of evolutionary conservation. Consequently it is apparent that as expected, only some hearing genes have been modified in bats during the evolution of echolocation, with others being subjected to purifying selection and perhaps being involved in more general aspects of audition rather than in specialized adaptations associated with echolocation. There is no evidence for positive selection acting on *Myo6* in echolocating bats (Shen et al., 2013), despite this gene being associated with hearing loss in humans (e.g., Oonk et al., 2013). Rather the gene is expressed at high levels in the kidneys of pteropodid bats, shows accelerated evolution in this lineage, and may have evolved in relation to the low protein intake from a frugivorous diet (Shen et al., 2013).

In summary, parallel evolution has been suggested for seven genes associated with a number of distinct auditory processing mechanisms in bats that use laryngeal echolocation. Although convergence seems a plausible explanation for similarities in genes seen between echolocating cetaceans and bats, is it really the case that convergent evolution has shaped the evolution of echolocation in yinpterochiroptean and yangochiropteran bats that use laryngeal echolocation? One evolutionary scenario is that the ancestor of all bats did not have the ability to echolocate, pteropodids never acquired it and that laryngeal echolocation convergently arose in the stem echolocating lineages. Another scenario is that laryngeal echolocation arose in the ancestor of all bats, convergently diversified in the extant echolocating lineages and was lost in the pteropodids (see Figure 2). A hypothesis of convergent gene evolution might predict that bats using tongue-clicking for echolocation (*Rousettus* species) would also have evolved convergent genetic mechanisms for auditory processing similar to those of laryngeal echolocators given the apparent sophistication of their biosonar (Yovel et al., 2011), although no such signatures have been seen.

Studies on gene convergence often emphasize that there is no evidence for relaxed selection acting on auditory genes in pteropodids that do not use laryngeal echolocation, which would suggest loss of echolocation capabilities, yet is an absence of relaxed selection in hearing genes truly indicative of loss of echolocation in pteropodids? Mammals rely heavily on hearing for survival; there is no non-pathogenic “deaf” phenotype observed in mammals (Kirwan et al., 2013). Therefore, the candidate “hearing” genes studied are under high purifying selection given that key mutations in these genes result in a deaf phenotype. True relaxed selection, which typically results in a loss-of-function mutation over time, should not be evolutionarily permissible. Therefore, given the conserved nature of these genes extensive relaxed selection should not be evidenced in pteropodids, even if echolocation capabilities were lost (Teeling et al., 2012; Kirwan et al., 2013).

In a recent comparative study of bat inner ear structures, Davies et al. (2013a) tackled this question of relaxed selection at the morphological level. The authors found that the cochleae of non-echolocating pteropodids showed little deviation from those of other non-echolocating mammals, whereas the cochleae of echolocating yinpterochiropterans and yangochiropterans were highly modified, and the latter showed evidence of a burst of morphological change following divergence of the two suborders. At the same time, this study revealed no clear support for a loss of echolocation in pteropodids. A related investigation of semi-circular canal morphology in echolocating bats found that the two major clades of echolocating species differed in canal size and shape in relation to body mass and cochlear size (Davies et al., 2013b). While these two studies cannot offer firm conclusions about whether laryngeal echolocation evolved more than once in bats, they do hint at independent evolutionary pathways consistent with multiple acquisitions.

How can the fossil record help inform our understanding of the evolution of echolocation? Whether or not the Eocene fossil bat *Onchonycteris finneyi*, dated at 52.5 Mya, was able to echolocate on the basis of anatomical traits has been the subject of considerable debate; in particular the small relative gross cochlea size suggests it could not (Simmons et al., 2008, 2010; Veselka et al., 2010). In contrast, Eocene fossil bats from other genera such as *Icaronycteris* and *Palaeochiropteryx* have been found to possess relatively larger cochleae that are indicative of echolocation capabilities (also see Simmons et al., 2008 and references within). If correct, the proposed placement of these echolocating genera on consecutive branches outside of the crown group of extant echolocating bats would necessitate further gains of echolocation (see Simmons and Geisler, 1998), a scenario that is arguably less parsimonious than a single loss in pteropodids. Such conflicting signals between molecular and morphological datasets regarding the issue of the evolution of echolocation highlight a need for more integrated approaches combining fossil evidence alongside molecular evolutionary analyses. In this regard, the recent and surprising finding that combined large-scale phenomic and gene datasets recover a monophyletic group of echolocating bats (O'Leary et al., 2013) warrants further study. Ultimately, a single origin of echolocation followed by secondary loss in pteropodids would be better supported if fossilized ancestral pteropodids with anatomical characteristics of echolocation were found, or if pseudogenization of genes known to be specific for echolocation could be identified in non-echolocating taxa (Teeling et al., 2012). This is challenging given that pteropodids have a poor fossil record that anatomical features may become damaged during fossilization, and also for the reason that genes associated with echolocation are likely to be variants of genes fundamental to vocalization and hearing in more general contexts. However, it is only through the integration of these different fields that the evolution of echolocation in bats will be elucidated.

## Vision

Vision is important for bats, especially for those bat species that do not echolocate. Vision can be effective over greater distances than echolocation and, although the latter provides more acuity (Suthers, 1970), bats use vision for orientation and for finding food (see review by Altringham and Fenton, 2003). Even in echolocating bats, prey detection may be multimodal, involving several senses (including vision), which are used according to perceptual constraints imposed by environmental conditions (Eklof and Jones, 2003). When vision and echolocation provide conflicting cues, visual cues are used preferentially (Chase, 1983; Orbach and Fenton, 2010). Recent research on the genetic mechanisms underpinning vision in bats has mainly focussed on the molecular evolution of light-sensitive pigments. These pigments consist of a membrane-bound G-protein-coupled receptor (an opsin) and a chromophore that undergoes photoisomerization when it absorbs light. Consequent conformational changes in the opsin result in transduction of signals, and thereby photons are transformed into electrochemical signals (Yokoyama and Yokoyama, 1996). Of course night vision has been understudied in bats, and is likely to involve a suite of adaptations in addition to opsin tuning. Hopefully some of the recent molecular evolutionary findings will inspire resurgence in research on behavioral aspects of vision in bats.

### Rods

Rods are the dominant photoreceptors in bat retinae (Suthers, 1970). Rods are adapted for vision in conditions where light levels are low, and are the main photoreceptors found in nocturnal mammals. The opsin in rods is known as rhodopsin, and its high sensitivity confers monochromatic vision under dim-light (scotopic) conditions. Zhao et al. (2009a) sequenced approximately 94% of the coding sequence of the rhodopsin (*Rh1*) gene from 15 bat species, and found that the gene was intact in all species studied. The authors determined the spectral tuning of rhodopsin from its amino acid structure. Wavelengths of maximum absorbance (λ_max_) were inferred as 497–501 nm, with most species having values at the upper extreme of this range (501 nm), fitting with the bats possessing the mammalian consensus compliment of critical amino acids. Rhodopsin has been under purifying selection during mammalian diversification, although rhinolophoid bats using high-duty cycle echolocation (species that emit CF signals with Doppler shift compensation) showed higher ratios of non-synonymous relative to synonymous mutations compared with other bats, perhaps as a consequence of relaxed selection (Zhao et al., 2009a).

Shen et al. (2010) amplified cDNA of *Rh1* from 15 bat species and recovered a different phylogenetic arrangement, with Pteropodidae forming a monophyletic group together with yangochiropterans to the exclusion of the yinpterochiropterans that use high-duty cycle echolocation. The authors argued that multiple incidences of convergent evolution in *Rh1* between yangochiropterans and pteropodids had occurred, though ecological factors that could have brought about such convergence are not clear. The same research team analysed evolutionary patterns in other genes involved in rod vision and adaptation to dimly lit conditions (Shen et al., 2012b). *Crx* is a photoreceptor-specific transcription factor involved in the differentiation of photoreceptor cells. *Sag* functions in desensitization of the photoactivated transduction cascade, and mutations in this gene can cause blindness at night in humans. Molecular signatures consistent with convergent evolution were detected in both genes, and was especially apparent in *Rh1* (two parallel changes in *Crx*, one in *Sag*) between pteropodid (Yinpterochiroptera) and emballonurid (Yangochiroptera) bats. The authors argued that the relatively large eyes found in both these groups of bats might utilize specialized rod-based visual mechanisms that resulted in convergent amino acid substitutions.

### Cones

Color vision in mammals is achieved in part by the possession of opsin proteins sensitive to short and medium- to long-wavelengths of light (Yokoyama and Yokoyama, 1996). Most living mammals are dichromatic and have a short-wavelength sensitive (Sws1—official name Opn1sw) opsin that is most sensitive to blue-violet wavelengths, and a medium- to long-wavelength sensitive (M/lws—official name Opn1mw) opsin with peak sensitivity in the red-green part of the spectrum (Peichl, 2005). Several lineages of nocturnal mammal species have lost function in *Sws1*, which has become pseudogenized, rendering color vision impossible (Jacobs, 2013).

**Figure 4:**
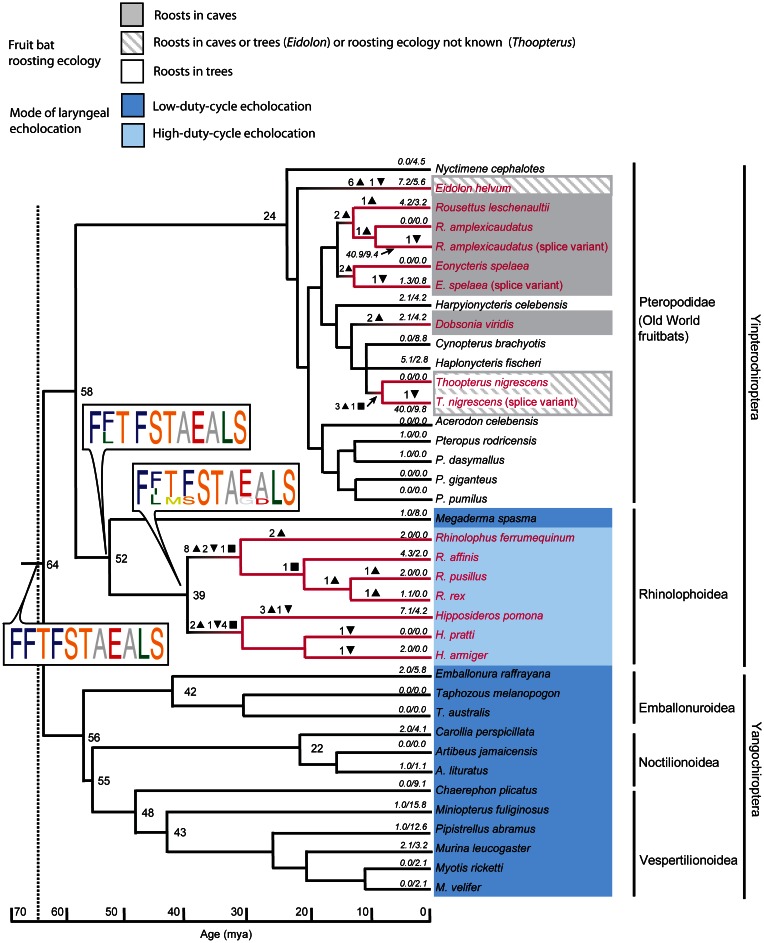
**Mutations in the short-wavelength opsin gene *SWS1* mapped onto the species tree based on published dated phylogenies of bats.** The tree shows substitution rates, indels, and stop codons. Numbers of insertions and deletions are illustrated by downward and upward triangles respectively. Inferred ancestral stop codons are shown by squares. Sequence logos show key changes in spectral tuning amino acid sites in which the height of the amino acid abbreviation is proportional to its posterior probability. Sequences with stop codons are shown in red font, with loss-of-function related to the presence of indels or stop codons illustrated by red branches. Branch lengths represent millions of years (MY), and numbers at nodes represent divergence times in MY. Numbers along terminal branches are ratios of non-synonymous to synonymous mutations after removing indels and stops. Note the loss of function associated with high-duty cycle echolocation and with cave roosting in pteropodids. From Zhao et al. (2009b), reproduced with permission from the National Academy of Sciences USA.

Zhao et al. (2009b) sequenced the *Sws1* gene in 32 bat species and the *M/lws* opsin gene in 14 species. Many bat species, like most diurnal mammals, appear at least potentially to be dichromats, with intact Sws1 and M/lws opsins. Why many nocturnal echolocating bats are potential dichromats deserves further research. Although the latter gene was conserved in all species studied, a loss-of-function of *Sws1* through pseudogenization was apparent in rhinolophoid bats that use high-duty cycle echolocation (i.e. species that use long CF signals and use DSC), and in some Old World fruit bats, especially in taxa that roost in caves (Figure 4). This loss-of-function appears to have arisen by independent genetic mechanisms in the ancestral nodes of the Hipposideridae and the Rhinolophidae, where stop codons or indels disrupted the open reading frame (ORF) of *Sws1* at different positions. Genetic evidence suggesting a loss of UV vision in bats with high-duty cycle echolocation and in cave-roosting pteropopids has also been supported by immunohistochemical evidence: after bats were stimulated with UV light, Fos-like expression in the primary visual cortex was more apparent in *Cynopterus sphinx* (a tree-roosting pteropodid) and *Scotophilus kuhlii* (uses low duty cycle echolocation) than in *Rousettus leschenaultii* (a cave roosting pteropodid) and *Hipposideros armiger* (uses high duty cycle echolocation) (Xuan et al., 2012).

Why all bats studied retained a functional M/lws opsin is unclear: perhaps the opsin may play a role in processes other than vision, for example the control of circadian rhythms (Zhao et al., 2009b). Ancestral reconstructions of amino acid sequences suggested that the ancestral vertebrate (and bat) short-wave opsin was ultraviolet (UV) sensitive, with a λ_max_ close to 360 nm. Because the Sws1 opsin has been under purifying selection in many bats, it could be that UV vision is important in many (mainly yangochiropteran) species. One phyllostomid (*Glossophaga soricina*) is indeed able to see UV stimuli, and UV signals may reflect strongly from flowers at low light levels (Winter et al., 2003). These recent findings on potentially functional opsins in bats should hopefully spur renewed interest in color vision in bats, and Zhao et al.'s (2009b) findings suggest that yangochiropterans should have better color discrimination abilities than rhinolophoid bats.

It is of interest that loss-of-function in *Sws1* occurs in bats with what is considered the most sophisticated type of biosonar known—high-duty cycle echolocation involving the emission of CF calls and Doppler shift compensation (Zhao et al., 2009b). This finding suggests that bats may be experiencing trade-offs associated with investment in the neural processing devoted to different senses. Such trade-offs have long been identified in investment in brain tissue (Harvey and Krebs, 1990) because of the extreme energetic demands imposed by neural processing (and even by signal production) (Niven and Laughlin, 2008). For example subterranean star-nosed moles show a reduction in the size of the visual cortex and an increase in the size of cortical regions associated with mechanosensory processing compared with the same parts of the brain in terrestrial hedgehogs (Catania, 2005). Obviously the development and maintenance of brain structures must have a genetic basis, and it is fascinating that potential trade-offs between vision and echolocation are now being identified through the process of pseudogenization leading to loss-of-function in sensory genes. Interestingly, the pseudogenization of *Sws1* for vision in the lineage of high duty cycle echolocators is also associated with accelerated evolution of *Prestin* for hearing in that lineage (Li et al., 2008; Zhao et al., 2009b).

## Olfaction

Olfaction is of great importance in the lives of bats. Frugivorous bats often use olfaction for finding food, and nectarivorous species can find flowers from scent cues. Furthermore, many bat species—perhaps all—use olfaction for communication including for mother-pup recognition, recognition of individuals and conspecifics. In some species for which olfaction appears to be of particular importance, specialized scent glands or tufts of hairs are used for the production and application of scent signals (see review by Altringham and Fenton, 2003).

Tetrapods possess two olfactory systems that have distinctive anatomical and neurophysiological bases (though potentially overlapping functions). All vertebrates studied to date, with the exception of some cetaceans (Kishida et al., 2007), possess a “main olfactory system” (MOS) for the detection of volatile stimuli. Smells are detected by olfactory sensory neurons in the olfactory epithelium in the nasal cavity. Olfactory sensory neurons send information to the main olfactory bulb in the brain, which in turn transmits information to the olfactory cortex and other brain regions. The Accessory Olfactory System (AOS) serves to detect fluid-based stimuli via a vomeronasal organ in the vomer (between the nose and the mouth). Nerve connections link the vomeronasal organ to the accessory olfactory bulb, and then signals are transmitted to the amygdala and the bed nucleus of the stria terminalis, and subsequently to the hypothalamus. Many tetrapods (including birds and many primates) lack an AOS, and the vomeronasal organ shows extensive variability in yangochiropteran bats (Bhatnagar, 1980). In a cladistic analysis of 18 bat families, Bhatnagar and Meisami (1998) concluded that the presence of a functional vomeronasal organ in phyllostomid bats, *Miniopterus* (Vespertilionidae) and *Pteronotus* (Mormoopidae) was the result of multiple gains, however, we suggest that multiple losses of an AOS is equally or more plausible.

### The main olfactory system

Olfactory receptors (ORs) are expressed in the cell membranes of olfactory sensory neurons located mainly in a small region of the upper nasal epithelium and initiate signal transduction cascades that send nerve impulses to the brain. They belong to the class A rhodopsin-like family of G protein-coupled receptors (Niimura and Nei, 2007). Each OR cell expresses only one odorant receptor, though each receptor can combine with several different odorants. Information from ORs is translated by the brain into a receptor code that represents a specific scent (Rinaldi, 2007).

In general, *OR* genes constitute the largest family of genes in the mammalian genome, for example comprising about 6% of the protein-coding genes in the dog (Lindblad-Toh et al., 2005; Hayden et al., 2010). There is enormous variability in the number of *OR* genes among mammal species—mice have approximately 1500 *OR* genes, humans about 800 (Niimura and Nei, 2003). Species that rely heavily on olfaction have large numbers of *OR* genes, whereas animals that specialize in using other senses have fewer functional *OR* genes, and typically high levels of pseudogenization. About half of the *OR* genes in humans are pseudogenes for example (Niimura and Nei, 2007). It is argued that a sensory trade-off exists between vision and olfaction in primates—with many *OR* genes becoming pseudogenized after primates evolved trichromatic color vision (Gilad et al., 2004). A high level of loss-of-function in *OR* genes is apparent in the platypus, which relies largely on mechanoreception and electrolocation for detecting prey, and in echolocating cetaceans (Niimura and Nei, 2007; Hayden et al., 2010).

Given that sensory trade-offs may have resulted in high rates of pseudogenization in other mammals that use specialized senses including electrolocation, echolocation, and trichromatic color vision, it is pertinent to ask whether high rates of pseudogenization are also apparent in echolocating bats. To address this question and explore the evolution of olfaction in bats Hayden et al. (2010) generated new *OR* gene sequence data (~2000 *OR* gene sequences) from aquatic mammals, semi-aquatic mammals, twelve bat species, and coupled these data with whole genome data from terrestrial mammals, resulting in ~50,000 OR gene sequences from 50 phylogenetically and ecologically diverse species. They analysed these data using a combination of phylogenetic, principal component, and Bayesian assignment tests, and identified unique signatures of OR gene family usage in bats. They uncovered spectacular examples of *OR* gene losses in three independent lineages of aquatic and semi-aquatic mammals, yet convergent, selective retention of similar functional *OR* families.

**Figure 5:**
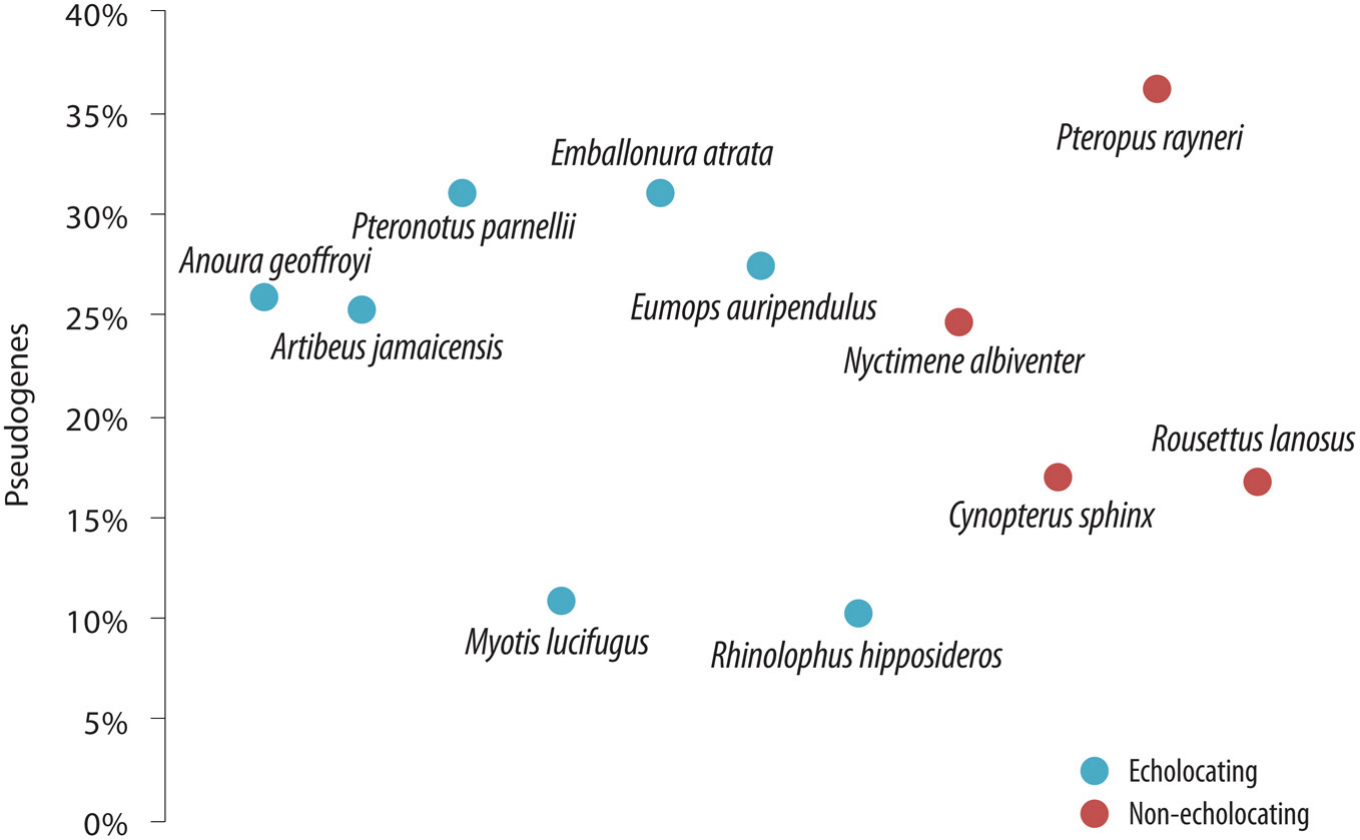
**The proportion of olfactory receptor (*OR*) genes that have become pseudogenes in a range of bat species that use laryngeal echolocation (blue symbols) compared with species that do not (red symbols).** From Hayden et al. (2010), reproduced with permission from Cold Spring Harbor Laboratory Press.

Despite the importance of echolocation in the lives of many bats, there was no evidence of a sensory-trade off resulting in extensive “death” of *OR* genes—bats appear to show similar percentages of pseudogenes (10–36%—relatively low levels for mammals in general) regardless of whether they use laryngeal echolocation or not (Hayden et al., 2010—see Figure 5). The percentage of *OR* genes that have become pseudogenes in bats is indeed unremarkable for mammals in general [cf. 28% in rat (Nei et al., 2008)], and lower than the ratio in humans (52%—Nei et al., 2008). Echolocating bats did not have more *OR* pseudogenes than non-echolocating bats. Indeed, the lesser horseshoe bat *Rhinolophus hipposideros* uses CF echolocation with Doppler-shift compensation, and only 10% of its *OR* genes are non-functional (Hayden et al., 2010). This species shows loss-of-function in the *SWS1* opsin gene (see above, Zhao et al., 2009b), and so perhaps a trade-off between color vision and echolocation has occurred, although olfaction has remained of importance in the life of this species. In comparison with other bat lineages the number of *OR* genes and the percentage of *OR* pseudogenes is quite low in rhinolophid bats, similar to the putative ancestral mammalian *OR* condition. This suggests that there was no massive “birth and death” of *OR* gene families in this species, most likely resulting from their long history of advanced echolocation capabilities, little reliance on olfaction for prey acquisition but a requirement of olfaction, most likely for communication. The fact that *R. hipposideros* possess olfactory genes that are mostly functional (90%), yet at the same time has a relatively small olfactory bulb (Neuweiler, 2000) could be seen as paradoxical. It follows that both genetic and anatomical data, together with information on the directionality of trait evolution, are all needed to reliably track the evolutionary history of sensory trade-offs.

### The accessory olfactory system

**Figure 6:**
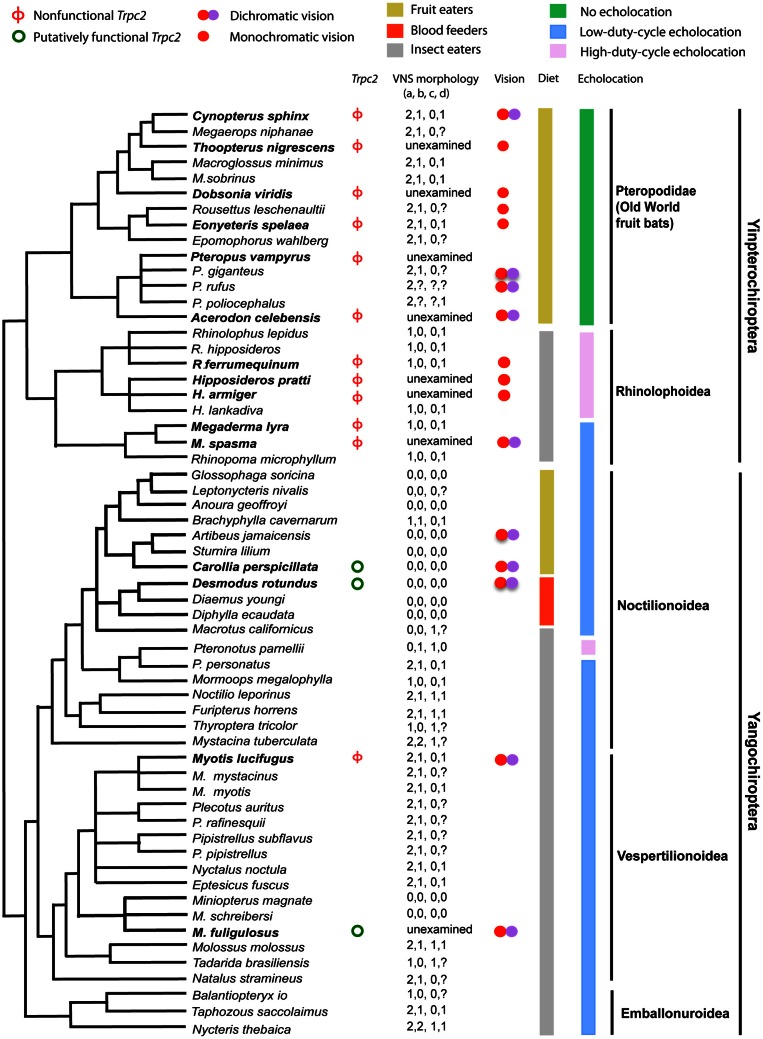
**Phylogenetic tree of bats for which information about the vomeronasal system (VNS) is available with information about *Trpc2* functionality, physiology and ecology.** Species in bold text had exon 2 of *Trpc2* sequenced by Zhao et al. (2011). VNS morphology—“a” represents vomeronasal epithelial tube (0 = well developed, 1 = rudimentary, 2 = absent), “b” is vomeronasal cartilage (0 = J, C, U, or O-shaped, 1 = bar-shaped, 2 = absent), “c” is information about the nasopalatine duct (0 = present, 1 = absent), and “d” refers to the accessory olfactory bulb (0 = present, 1 = absent). Missing data are coded by “?”. Note the limited functionality in *Trpc2*, that genetic functionality corresponds with anatomical functionality, and that functionality occurs in two divergent lineages of bats, suggesting multiple many losses of the VNS across the order of bats. From Zhao et al. (2011) and reproduced with permission from Oxford University Press.

There is also some evidence in support of sensory trade-offs affecting the vomeronasal system in tetrapods, as it has been lost in primates with trichromatic color vision and in birds with tetrachromatic color vision (Zhang and Webb, 2003). *Trpc2* is a gene that can be used to determine vomeronasal sensitivity as it is essential for vomeronasal signal transduction and has no known alternative function (Grus and Zhang, 2006). Zhao et al. (2011) sequenced the longest exon (exon 2) of *Trpc2* from 13 bat species and found widespread loss-of-function (Figure 6). Multiple indels and premature stop codons were identified in all 10 yinpterochiropterans studied, with some suggestion of independent loss-of-function in Pteropodidae and Rhinolophoidea. Three yangochiropterans studied—*Miniopterus fuliginosus* (Miniopteridae), *Carollia perspicillata* and *Desmodus rotundus* (Phyllostomidae) showed intact exon 2 ORFs and the sequence was under purifying selection (Zhao et al., 2011). Examination of draft genome sequences for *Pteropus vampyrus* and *Myotis lucifugus* suggested that *Trpc2* had been pseudogenized in both species independently (Zhao et al., 2011). These findings are consistent with the anatomical findings of Bhatnagar and Meisami (1998) who reported functional vomeronasal organs in phyllostomid bats and *Miniopterus*, and only otherwise in *Pteronotus* among other bats from 18 families examined.

The extensive loss-of-function of the vomeronasal system in bats does not appear to be related to sensory-trade offs in any obvious way. Loss-of-function is apparent in echolocating and non-echolocating taxa, in dichromatic and monochromatic species, and is not related to the amount of pseudogenization in *OR* genes (Zhao et al., 2011). The only limited evidence for a trade-off occurs in vampire bats, which show loss-of-function in a sweet taste receptor gene but possess a functional vomeronasal system (Zhao et al., 2010a).

## Taste

Taste, or gustation, results from sensations produced when substances react with taste bud receptors in the mouth. There are five primary tastes—sweet, bitter, umami, salty and sour. Genes involved in the last two of these have not been studied in bats. Sweet, umami and bitter are sensed via molecules binding to G protein-coupled receptors (GPCRs) found on the cell membranes of taste buds.

### Sweetness

Sweetness is useful for the detection of energy-rich foods such as sugars. A family of GPCRs known as Tas1rs functions in the detection of sweet and umami tastes. Only three *Tas1r* genes have been described in mammals, with the Tas1r2 and Tas1r3 heterodimer functioning in the detection of sweetness, and the Tas1r1 and Tas1r3 heterodimer functioning as the umami taste receptor. Hence Tas1r2 is thought to be the only taste receptor specific to sweetness, and *Tas1r2* knockout mice show disrupted responses to sweet taste (Zhao et al., 2003).

Zhao et al. (2010a) sequenced approximately 720 bp of exon 6 from *Tas1r2* in 42 bat species representing a wide range of families and dietary habits. *Tas1r2* evolved in the common ancestor of bony vertebrates, and the sequence analysed has remained conserved and under purifying selection in all bat species studies except for three species of sanguivorous vampire bats (Zhao et al., 2010a). The highly specialized diet of these bats has presumably made the need to discriminate among tastes redundant. Pseudogenization of *Tas1r2* in the three vampire bat species involved different ORF-disrupting mutations, though the relaxation of functional constraints may have already occurred in their common ancestor and the mutations documented in the relatively short portion of *Tas1r2* examined may have been the consequence of neutral evolution following an earlier pseudogenization event that preceded the evolution of sanguivory (Zhao et al., 2010a).

### Umami

Umami is an appetitive taste, and humans perceive savory or meat-like tastes via umami receptors. Umami may function in the detection of amino acids that may signal nutritious food (Herness and Gilbertson, 1999). Using the same logic as described above for *Tas1r2*, Zhao et al. (2012) sequenced a portion of *Tas1r1* as a probe for the ability to taste umami in bats. Previous studies had shown the gene to be intact in all mammals studied except the giant panda (Zhao et al., 2010b). However, *Tas1r1* was absent, not amplifiable, or pseudogenized in all of 31 bat species studied, implying that the umami taste may have been lost in bats. Why bats—that exploit a wide variety of diets—do not need umami is unclear.

Vampire bats are especially interesting because all three of their Tas1rs appear to be non-functional (Zhao et al., 2012). Vampire bats are therefore unable to taste sweet or umami, and this fits with the lack of ability of common vampire bats *Desmodus rotundus* to learn aversions to harmful foods (Ratcliffe et al., 2003), and their indifference to high sugar concentrations (Thompson et al., 1982). Vampire bats are the only mammals so far known to lack two tastes. It is tempting to speculate that this represents a sensory trade-off with their functional vomeronasal systems and use of infrared heat sensing, though Zhao et al. (2010a) argue that the loss-of-function in Tas1r1 predated the origin of vampire bats. Whether it predated the evolution of sanguivory is of course debatable.

### Bitter taste

The ability to detect bitter tastes is likely to be adaptive because bitterness is often associated with harmful food items. Whereas the likely consequences arising from molecular evolutionary patterns in sweet and umami tastes are relatively easy to predict because each the GPCRs involved is encoded by a single gene (Shi and Zhang, 2006), the situation regarding bitter taste is more complex. Taste receptors known as T2Rs are responsible for sensing bitterness. Although bitter taste receptors are also GPCRs, *T2R* gene repertoires are extremely variable among species, and as is the case for *OR* genes, evolved by extensive gene duplication and birth-and-death evolution that result in extensive gains and losses of *T2R* genes in all lineages of mammals studied (Dong et al., 2009). Zhuo et al. (2009) examined the *T2R* repertoire in the draft, relatively low coverage (1.7×) genome of the insectivorous little brown bat *Myotis lucifugus*. Twenty-eight *T2R* genes were detected in the bat genome, of which nine appeared intact, eight partial but perhaps still functional, and nine were pseudogenes. This compared with 37 functional genes and 11 pseudogenes in humans, and 37 functional genes and five pseudogenes in the rat. One clade of bat-specific genes was identified, implying that bitter tastants specific to bats may have evolved. Strong positive selection had shaped the evolution of the *T2R* gene repertoire in bats (Zhuo et al., 2009).

## Thermoperception

The common vampire bat *Desmodus rotundus* is the only mammal known to possess heat-sensing organs. These bats have three 1-mm diameter pits situated between nasal pads and the noseleaf that are maintained at a cooler temperature than other areas on the face, and are used for the detection of warm temperatures on endothermic prey items that the bats extract blood meals from (Kürten and Schmidt, 1982). Similar structures may exist on the two other species of vampire bats (Altringham and Fenton, 2003).

Vampire bats detect infrared signals by trigeminal nerves that innervate the pit organs in ways that are in some respects convergent with but in other ways radically different from mechanisms of infrared detection by boas, pythons and pit vipers (Kürten et al., 1984; Gracheva et al., 2011). Although both groups use pit organs in the face (albeit in different regions) that are innervated by trigeminal nerves for heat detection, the heat-sensitive channels used by snakes and vampire bats for infrared detection differ significantly. Snakes modify a non-heat sensitive channel (the transient receptor potential A1 or TRPA1 channel) as an infrared detector (Gracheva et al., 2010). Vampire bats produce a shorter version of another member of the TRP family, TRPV1, which includes a small exon that contains a stop codon, by alternative splicing. Alternative splicing can generate a range of distinct RNA variants and consequently proteins with different functions from a single mRNA precursor by the differential joining of 5′ and 3′ splice sites. Gracheva et al. (2011) used an experimental approach—expressing the novel short version of TRPV1 from vampire bats in *Xenopus* oocytes and performing electrophysiological assays—to show the shorter version of the protein is activated at 30°C. Hence the vampire bats maintain the original function of the TRPV1 channel—noxious heat detection at temperatures >43°C, while also obtaining a novel ability to detect body heat for the detection of vital blood meals via the short variant of the protein. This study highlights how thermoperception can arise through mechanisms that involve similar nerve pathways but involve different molecular mechanisms, and illustrates the importance of alternative splicing in the evolution of novel adaptations.

## The future

Studies to date on the molecular basis of sensory biology in bats have focussed on determining patterns of molecular evolution in candidate genes that have known functions in humans and other model organisms. Often these genes have been targeted because of studies that detected phenotypic defects in humans resulting from mutations, as is the case with genes associated with vocalizations (e.g., dysphasia and dyspraxia resulting from mutations in *FOXP2*) and hearing (e.g., non-syndromic deafness resulting from mutations in hearing genes). Advances in transcriptomics and whole genome sequencing will allow genomic comparisons between mammals with different sensory abilities to be performed at a much larger scale and potentially identify novel genomic regions under sensory selection in bats. Next generation sequencing is making it increasingly possible to identify genetic loci responsible for adaptive evolution in non-model organisms, and the field of adaptation genomics holds great promise (Stapley et al., 2010; Hughes et al., 2013; Zhang et al., 2013).

Differences in gene regulation in bats have been little explored to date. These are likely to be important—for example replacement of the endogenous mouse *Prx1* gene regulatory element with the bat homologue causes limb elongation in mouse embryos by increasing Prx1 expression in the perichondrium, leading ultimately to longer forelimbs in the mice (Cretekos et al., 2008). Differences in patterns and the timing of gene expression, rather than solely changes in the genes themselves may play a major role in the evolution of sensory performance in bats, and yet studies on gene expression and on regulatory genes associated with sensation in bats are still in their infancy.

The importance of alternative splicing in generating proteomic diversity in bats remains largely unknown. Between 40 and 60% of human genes have alternative splice forms, and these comprise one of the major components of functional complexity in the proteomes of humans and other mammals (Modrek and Lee, 2002; Keren et al., 2010). For example, isoforms of the Slo protein expressed in the rat cochlea vary in deactivation kinetics and Ca^2+^ sensitivity, and their occurrence is partly determined by hormonal stress (Xie and McCobb, 1998). The importance of splice variants in bats remains largely unknown; however, Li et al. (2008) identified alternative splice forms of the *Prestin* gene in bat brain and cochlea tissue. Such isoforms might be expected to produce a range of functional outcomes from genes associated with audition in bats. Similarly the importance of other processes contributing toward functional diversity, such as RNA editing (e.g., Garrett and Rosenthal, 2012), is not known for bats and other mammals.

Studies on molecular evolution suggest major differences in the sensory performance of different bat lineages, and set a platform for exciting behavioral experiments. For example, the loss of function of *Sws1* in rhinolophoid bats suggests that these bats should be unable to perceive short wavelengths of light, yet yangochiropterans are dichromats and should have retained this ability. Although we do not know for sure whether intact genes result in the ability to detect short wavelengths (physiological features in the lens may for example influence this), the hypothesis that rhinolophoid and yangochiropteran bats show different abilities in their detection and discrimination between different wavelengths of light seems ripe for testing. Given that bats with intact vomeronasal signal transduction genes are indeed those species known to have functional vomeronasal systems, and that bats with pseudogenized sweet and umami taste receptors are unable to learn taste aversions suggests that linking the genetic basis of sensory behavior to sensory performance has great promise. Research on the sense of touch might also be illuminating. The recent discovery that tactile receptors on bat wings are sensitive to airflow (Sterbing-D'Angelo et al., 2011) makes unraveling genetic mechanisms underpinning the tactile sense in bats an interesting challenge.

### Conflict of interest statement

The authors declare that the research was conducted in the absence of any commercial or financial relationships that could be construed as a potential conflict of interest.
